# Artificial Intelligence for Infant Pain Detection: A Bibliometric and Knowledge Mapping Review

**DOI:** 10.3390/bioengineering13101124

**Published:** 2026-09-26

**Authors:** Wan Azani Mustafa, Norhayati Mohd Zain, Halimaton Hakimi, Muhammad Naufal Mansor, Debrina Puspita Andriani

**Affiliations:** 1 Advanced Computing Engineering (AdvComp), Centre of Excellence (CoE), Pauh Putra Campus, Universiti Malaysia Perlis (UniMAP), Arau 02600, Perlis, Malaysia; 2 Faculty of Electrical Engineering Technology, Pauh Putra Campus, Universiti Malaysia Perlis, Arau 02600, Perlis, Malaysia; 3 Medical Imaging Department, School of Integrative Medicine & Life Sciences, KPJ Healthcare University, Nilai 71800, Negeri Sembilan, Malaysia; norhayati@kpju.edu.my; 4 Fellowship, O.P. Jindal Global University, Department of Computing, Universiti Teknologi PETRONAS, Seri Iskandar 32610, Perak, Malaysia; 5 Department of Industrial Engineering, Faculty of Engineering, Universitas Brawijaya, Malang 65145, Indonesia; debrina@ub.ac.id

**Keywords:** artificial intelligence, infant pain detection, bibliometric analysis, science mapping, biblioSpy, neonatal care

## Abstract

Artificial Intelligence (AI) has been created to be a potent tool that can help clinical decision-making by offering objective and ongoing monitoring of neonatal health. Unlike adults, infants are unable to communicate pain verbally and conventional pain measurement relies primarily on subjective and observational pain scales. This dependence introduces the possibility of some interrater variability and observer bias that may result in variability in assessment and suboptimal pain management in neonatal intensive care settings. To address these challenges, this study aims to provide a complete overview of the literature on AI-based infant pain detection and draw an intellectual map of this field by conducting a comprehensive bibliometric and science-mapping review. The systematic search was carried out by employing the Scopus database with an advanced and structured keyword search query to retrieve relevant English-language journal articles from 2010 to 2026. After screening with predefined inclusion criteria, a final set of 153 core documents was retained for analysis. The research productivity and citation impacts of the identified articles were assessed, and emerging thematic structures were identified through bibliometric processing and network visualization using a web-based bibliometric analysis platform, BiblioSpy^®^. Quantitative results demonstrate that the field is dynamic and expanding rapidly, with an annual publication growth rate of 29.06% and 2474 cumulative citations. The h-index of the analyzed corpus is 26, and the g-index is 46, with an average of 6.08 authors per publication and an 88.24% international co-authorship rate, suggesting a very collaborative environment. To conclude, quantitative keyword temporal tracking confirms this evolution: traditional binary algorithms display earlier average publication timelines (2019.0), whereas deep learning, Convolutional Neural Networks (CNNs), and multi-signal predictive models dominate recent outputs (2023.8–2024.5), demonstrating a quantitative shift from isolated binary classifiers toward continuous, explainable, and clinically viable multimodal monitoring systems. The results indicate that although the initial studies have been done on only binary classification of facial expressions and/or cry acoustics, the current research is moving towards continuous, explainable, and clinically viable multimodal monitoring systems.

## 1. Introduction

The use of AI has become a game-changer in the healthcare industry, especially in the context of pain recognition in newborns. Infants are unable to express pain verbally, and traditional assessment tools rely on subjective observations of behavioral and physiological indicators. These approaches are not always reliable or time-efficient and may result in suboptimal pain management [1,2,3]. AI holds the potential to provide an alternative solution, as it can employ advanced techniques such as facial expression analysis and audio signal processing while integrating multimodal data to enable the automated, objective, and efficient detection of pain. CNNs and transfer learning models have been identified as highly accurate in recognizing various facial expressions associated with pain, with some of the models achieving up to 95% accuracy [4]. Such advancements not only contribute to more accurate pain assessment but also facilitate timely interventions to enhance the quality of care for neonates [5].

However, several hurdles must be overcome in the implementation of AI in clinical practice. Ethical concerns, such as the importance of using AI in a manner that augments rather than replaces human decision-making, remain a key consideration [6]. Additionally, challenges related to obtaining high-quality datasets and accounting for variations in lighting conditions, movement, and partial face occlusion must be addressed to ensure consistent performance across diverse clinical scenarios [7]. While stakeholders have expressed positive views about the potential of AI, they have also raised concerns about the need for human oversight and the importance of addressing data privacy and accessibility issues. In summary, AI presents considerable potential for improving the detection of infant pain, but its challenges and limitations must be addressed through a multidisciplinary approach, with ethical and practical considerations remaining at the forefront of neonatal care [8].

## 2. Literature Review

AI-based infant pain detection is an emerging research field focused on advancing infant pain assessment towards more objective, continuous, and clinically applicable methods. Infants are unable to verbally report pain, and traditional instruments rely on observations of infants’ behavior and physiology, which are subjective, inter-rater-dependent, and time-consuming to administer in neonates [9]. These restrictions are particularly relevant because neonates in intensive care frequently undergo painful procedures, while access to effective analgesia is often restricted; thus, there is a need for accurate and timely recognition of pain. In the literature, there are three key types of signals used to develop AI systems: (1) Facial expressions, (2) Cry acoustics, and (3) Physiological measurements, with a trend towards multimodal systems that integrate all these types of signals for better, more clinically robust predictions [10,11].

Facial-expression analysis is the most established AI approach for neonatal pain assessment because facial activity is considered a highly specific pain indicator in nonverbal infants [10,12]. Early and contemporary studies have used static images and video to classify pain versus no pain or to grade pain intensity. The Classification of Pain Expressions (COPE) project is identified as the first published machine learning study on infant pain evaluation, using facial images and classifiers such as principal component analysis, linear discriminant analysis, and Support Vector Machines (SVMs). More recent deep learning studies report improved performance: a CNN trained on the COPE database achieved 90.24% test accuracy with an Area Under the Curve (AUC) of 0.9818. However, the authors explicitly note the small sample size and the need for external validation [13]. In a real-world neonatology setting, a fine-tuned pretrained CNN applied to heel-prick images achieved an 87.4% average accuracy and a 75.4% F1 score. Performance improved further when iCOPE images were added to the training dataset [14]. Video models add temporal information that static images cannot capture; for example, cross-stream attention built on a two-stream convolutional network improved four-class neonatal pain-expression recognition on the DFEPN video dataset. Nevertheless, overall four-class accuracy remained modest at 66.20%, indicating that multi-level pain grading is harder than binary pain detection [12].

A second major line of work treats infant crying as the primary signal through which pain and other needs can be inferred. Most studies on cry-analysis transform audio signals into spectrogram-like or cepstrum-like representations and use classical machine learning, deep learning, or a combination of both approaches [15,16]. CNN-extracted spectrogram features were also used with an SVM to achieve 88.89% accuracy in neonatal cry classification, suggesting the effectiveness of deep feature extraction in moderate-size datasets rather than massive datasets [17]. Other studies indicate even better performance for more general cry recognition tasks: one study reported a 96.39% accuracy rate for random forest classifiers trained using MFCC, ZCR, and RMS features, and more recently, CNNs and vision transformers in cry recognition for needs-related cries achieved an accuracy rate of 98.33% [18]. Results of systematic reviews of cry analysis suggest that machine learning can distinguish pathological and need-related cries; however, accuracy is variable, ranging from 44.5% to 99.82% across the different data sets, tasks, and feature sets [19]. Nevertheless, one of the main limitations of pain-specific interpretations is that while cry acoustics can indicate pain, they can also indicate hunger, discomfort, tiredness, burping, and distress, meaning that a great performance on curated datasets does not necessarily ensure an unambiguous detection of pain in noisy real-world settings [20].

The field is now transitioning from single-modality classification to clinically oriented, multimodal, and real-time systems. Multimodal deep learning, combining infant face and voice features, has performed better than single-modality models. One study found that voice features were more relevant than facial features alone, and that combining features resulted in better scores than either input alone [11]. The same study suggested that it would be useful to consider adding other physiological parameters, such as heart rate, breathing pattern, and oxygenation, as these measures may indicate more evidence of severe stimuli when facial or cry patterns are not as informative [11]. This is now becoming a reality, as a post-operative NICU system that combines heart rate, respiration, oxygen saturation, and computer vision has been reported to achieve 74% AUC and 67.59% mAP for automatic pain detection, with an early pain detection module able to predict pain onset approximately 5–10 min before it occurred [21]. EEG, near-infrared spectroscopy, facial expression, body movement, oxygenation, and heart rate are among the other evidence sources that have been integrated with voice recordings to benefit the classification of distress; a CNN achieved 93% accuracy and revealed correlations between acoustic, neural, and physiological evidence [22]. Thus, these results corroborate the general agreement that multimodal monitoring better reflects clinical pain assessment and is better suited for continuous monitoring in NICUs [9,19].

Apart from the classification accuracy, robustness, interpretability, datasets, and deployment constraints are also important factors for clinical translation. Multiple studies indicate that face detectors trained on adults or acquired from the literature do not perform well on NICU images due to significant differences in occlusion, lighting, pose, and neonatal facial proportions; one study found that a neonatal-specific YOLO detector increased mAP by 8.6% and AUC by 21.2% compared to manual-scoring workflows [21]. Explainability has also become a focus: one newborn facial-pain model employed visual explanation techniques to confirm that predictions were based on facial parts related to pain; another study suggested a continuous, interpretable “pain sign” instead of binary categories and classified trajectories as stable, irregular, unstable, or indeterminate, depending on their clinical utility [23]. Another significant improvement is the growth of the datasets. FENP contains 11,000 neonatal images with four classes: severe pain, mild pain, crying, and calmness; whereas the newer IFEPI contains 25,000 spontaneous images from 120 infants in four pain categories, with the best test accuracy ranging from around 94.6% to 95.0% for the top deep models [24,25]. Meanwhile, numerous papers continue to focus on the challenges of small data sets, annotation challenges, noise sensitivity, and limited external validation as key factors to tackle in order to achieve generalizability [13]. Overall, the literature reveals that AI has the potential to accurately detect baby pain (with promising accuracy in particular for facial expression and cry signals). The most promising direction is towards multimodal, continuous, and explainable monitoring, rather than isolated binary classifiers. The next major hurdle is whether AI systems can perform well across different hospitals, devices, and patient groups, and be reliable tools for use in routine neonatal care [14,16,23].

## 3. Research Questions

Accordingly, this study addresses the following research questions:

RQ1. What are the key bibliographic characteristics of the literature on AI-based infant pain detection?

RQ2. How has the scientific output on AI-based infant pain detection evolved?

RQ3. Which journals constitute the core publication sources in this research field?

RQ4. Which authors have made the most influential scholarly contributions to this field?

RQ5. What is the relationship between author productivity and citation impact in this research domain?

RQ6. What are the global patterns of scientific contributions, and which geographical regions remain underrepresented?

RQ7. What are the dominant research themes and emerging knowledge structures in AI-based infant pain detection?

RQ8. What are the patterns of international research collaboration in AI-based infant pain detection, as revealed through country co-authorship networks?

## 4. Materials and Methods

The bibliometric analysis employed an integrated workflow combining data preprocessing, performance analysis, and science mapping to enhance methodological transparency and reproducibility. Bibliographic records were first subjected to data cleaning and harmonization using OpenRefine version 3.8.2, including deduplication and standardization of author names, institutional affiliations, and author keywords. Spelling and formatting inconsistencies were resolved to minimize artificial fragmentation of equivalent entities and terms. Microsoft Excel was subsequently used for descriptive statistical analysis and the tabulation of bibliometric indicators, including publication trends, citation metrics, source productivity, author productivity, and institutional and country contributions. Science-mapping and network analyses were conducted using VOSviewer version 1.6.21. Keyword co-occurrence analysis was performed using a minimum occurrence threshold of five occurrences, with full counting selected as the counting method. Association-strength normalization was applied to normalize the strength of relationships between items. Network mapping was performed using the VOS mapping technique, while clusters of related items were identified using the VOS clustering technique. The standard VOSviewer layout parameters were retained without additional manual modification to maintain consistency and reproducibility across the network visualizations. The same analytical framework was applied to the construction and visualization of country collaboration networks.

BiblioSpy^®^ was employed as an integrated web-based bibliometric platform to organize bibliographic records, facilitate data management, generate descriptive bibliometric summaries, and support the reporting and presentation of bibliometric outputs. In this study, BiblioSpy^®^ was primarily used to generate and organize the overall bibliometric profile of the dataset, including publication trends, citation indicators, source and author productivity, and institutional and country contributions. It was not used as the sole analytical tool for the science-mapping analyses. In particular, the keyword co-occurrence and country collaboration networks reported in this study were constructed and visualized using VOSviewer. This distinction was made to ensure that the core network-analysis procedures were based on an established and independently reproducible science-mapping platform. The integration of descriptive bibliometric indicators with network-based science mapping enabled the examination of the field from complementary perspectives, encompassing research performance, intellectual structure, social collaboration, and thematic development. This multi-stage analytical workflow provided a systematic basis for characterizing the evolution and knowledge structure of research on AI-based infant pain detection while improving the transparency and reproducibility of the analytical procedures.

### Data Search Strategy

The present study was designed as a bibliometric and knowledge-mapping analysis rather than a systematic assessment of methodological quality or clinical effectiveness. Accordingly, journal quartile, publisher-level ranking, and study-level quality scores were not used as additional inclusion or exclusion criteria. Eligible publications were identified from the Scopus database according to the predefined search strategy and established inclusion and exclusion criteria. The complete eligible corpus was retained to minimize selection bias and to provide a comprehensive representation of the development, thematic structure, citation patterns, and collaboration landscape of AI-based infant pain detection. Importantly, Scopus indexing was used as a bibliographic database criterion and should not be interpreted as evidence that all included publications are of equivalent methodological rigor, clinical relevance, or scientific impact.

A systematic and reproducible literature search and screening protocol was developed to identify publications relevant to AI-based pain detection in nonverbal infants. The search strategy was designed to maximize conceptual coverage while minimizing the risk of selection bias associated with inconsistent terminology across the biomedical and computer science literature. Search terms were derived from the core concepts identified in the relevant domain literature and expanded to incorporate common synonyms and terminology used across research on neonatal pain assessment, AI, and medical imaging or signal-processing methods. The search strategy was structured around four conceptual dimensions: population (baby, infant, newborn); clinical condition (pain, discomfort, suffering, distress); technical task (detect, detection, recognition, identification); and enabling technologies (AI, machine learning, and deep learning). Where applicable, terminology was cross-checked against established biomedical vocabularies, including Medical Subject Headings (MeSH), to enhance synonym coverage and reduce the risk of overlooking studies using alternative terminology. The predefined query was applied systematically, and all records retrieved by the automated search that satisfied the specified inclusion criteria were retained for bibliometric analysis, thereby minimizing discretionary selection during corpus construction. The use of broader umbrella terms, particularly distress alongside pain and newborn alongside infant, was intended to capture the heterogeneous terminology used to describe non-verbal pain- and distress-related signals in early life. This intentionally inclusive strategy also resulted in the retrieval of a small number of conceptually peripheral publications, including studies addressing broader perinatal distress, neonatal pharmaceutical care, and foundational computer-vision methods for medical imaging. These records were retained within the original bibliometric corpus because they were identified through the predefined search strategy rather than being selected on the basis of citation prominence. Their presence should therefore be interpreted as reflecting the broader methodological and conceptual landscape surrounding AI-enabled infant assessment, rather than as evidence that each publication directly investigated infant pain detection.

The resulting search strategy was applied to the TITLE-ABS-KEY fields of the Scopus database, allowing the retrieval of studies in which the relevant concepts appeared in the title, abstract, or author-supplied keywords. Boolean operators and truncation were used where appropriate to combine related terms within and across the four conceptual dimensions. Following the initial retrieval, predefined eligibility criteria were applied to establish a consistent analytical corpus. The inclusion criteria comprised English-language, peer-reviewed journal articles published at the final publication stage between 2010 and June 2026. Conference proceedings, editorials, book chapters, notes, letters, and preprints were excluded. Restricting the corpus to final peer-reviewed journal articles was intended to provide a consistent publication type and a relatively stable bibliographic record for subsequent bibliometric analysis, while excluding document categories with substantially different publication and indexing characteristics. The English-language restriction was applied to maintain consistency in data processing and terminology normalization, particularly for keyword co-occurrence and thematic mapping. The search period from 2010 to June 2026 was selected to capture the development of AI-enabled approaches to infant pain assessment from the emergence of computational methods through the most recent literature available at the time of data retrieval. Next, the screening procedure was executed in two distinct phases. In Phase 1 (Title and Abstract Screening), two reviewers independently evaluated all 239 initial records retrieved from Scopus against the eligibility criteria. Records that clearly met exclusion criteria were eliminated. In Phase 2 (Full-Text Evaluation), the remaining full-text articles were independently examined by both reviewers, with clinical validation overseen by a senior specialist in medical imaging and pediatric care. Inter-reviewer disagreements regarding eligibility during both phases were systematically addressed through structured consensus meetings. If consensus was not immediately reached, a third senior author reviewed the relevant document to make a final determination. This screening process resulted in the exclusion of 86 records due to scope mismatch, yielding a final analytical corpus of 153 core journal articles. The complete search and screening procedure was subsequently used to establish the final dataset for bibliometric performance analysis, collaboration analysis, and science mapping.

A total of 239 records were retrieved from Scopus using the predefined search strategy. The retrieved records were subsequently screened against the study’s prespecified inclusion and exclusion criteria, which were developed in accordance with the research objectives and scope. Following the initial screening, 86 records were excluded because they did not adequately align with the defined scope or research focus, resulting in a corpus of 153 records for bibliometric analysis. This systematic screening process was undertaken to ensure that the analytical corpus was sufficiently aligned with the conceptual domain of AI-driven infant pain assessment while maintaining transparency and reproducibility throughout the study selection process.

To further assess topical alignment, the resulting 153-record corpus underwent a secondary screening by two independent reviewers, with discrepancies and methodological considerations overseen by a senior specialist in clinical medical imaging. Rather than selectively removing records with broader topical relevance after the search process, potentially relevant cross-disciplinary publications were retained in the bibliometric dataset where they satisfied the predefined eligibility criteria. This approach is consistent with the principles of bibliometric science mapping, in which retaining the retrieved and eligible corpus supports search-strategy reproducibility, minimizes post hoc selection bias, and enables a more representative examination of the intellectual structure of an emerging interdisciplinary field. In particular, peripheral contributions involving general image-texture descriptors, physiological or behavioral indicators, and perinatal stress monitoring may provide important methodological links to the broader development of AI approaches for infant pain assessment. Accordingly, the final corpus of 153 documents was used for subsequent bibliometric analysis and science mapping. The overall process and associated steps are illustrated in Figure 1.

## 5. Results and Findings

RQ1. What are the key bibliographic characteristics of the literature on AI-based infant pain detection?

The bibliometric characteristics of research on AI-based infant pain detection reveal that the field is rapidly growing and maturing. From 2010 to 2026 (17 years), a total of 153 publications were identified, and these publications received 2474 citations (Table 1). The annual growth rate of 29.06% indicates a strong and sustained scholarly interest in the field. This growth indicates increasing research attention to the application of AI in neonatal healthcare. The mean age of the documents (2.74 years) also indicates that a large proportion of the documents are recent, further highlighting the relative newness of the field. The literature is relatively young, but is moderately to highly academically influenced, with an average of 16.17 citations per document and 20.79 citations per cited paper.

The data set also indicates a very collaborative research environment. A total of 930 author contributions were identified, representing the 854 unique authors that contributed to the publication of the identified studies. Four of these documents had only one author, and the average number of authors per document was 6.08. This trend is consistent with the interdisciplinary nature of AI infant pain detection, which requires expertise in computer science, biomedical engineering, neonatology, nursing, psychology, and data science. The involvement of 411 institutions in 55 countries also reflects the international and multidisciplinary nature of the field. In particular, the high percentage of international co-authorship (88.24%) indicates that this is increasingly a way of knowledge production, allowing researchers to draw on complementary clinical skills, technological capabilities, and datasets.

Beyond publication volume, citation-based indicators provide complementary evidence of the intellectual development and scholarly attention received by the field. The analyzed corpus yielded an h-index of 26 and a g-index of 46. An h-index of 26 indicates that 26 publications within the corpus have each received at least 26 citations, identifying a core body of frequently cited literature. The higher g-index of 46 indicates that a subset of publications has accumulated a disproportionately high number of citations relative to the broader corpus. These indicators should, however, be interpreted in relation to the relatively young age of the literature, with an average document age of only 2.74 years. Consequently, the observed citation profile reflects an emerging knowledge base in which opportunities for longer-term citation accumulation remain limited. The m-index of 1.529 provides additional temporal context by relating the h-index to the duration of the research activity represented in the corpus. Rather than interpreting this value as an absolute measure of research quality, it indicates that citation accumulation has developed within a comparatively short period. This pattern is consistent with the field’s rapid expansion. Taken together, these indicators suggest that research on AI-based infant pain detection is progressing beyond isolated methodological investigations toward a more established research domain. The 1759 citations accumulated within the h-core further demonstrate that scholarly attention is concentrated within a defined group of highly cited publications. Collectively, the h-index, g-index, m-index, and h-core citation count provide complementary perspectives on the field’s citation structure and temporal development rather than serving as standalone measures of research quality.

Contextual comparison with broader healthcare AI research further illustrates the specialized nature of this literature. Broader and more mature healthcare AI domains generally encompass substantially larger publication volumes and longer citation histories than the present corpus, and therefore their absolute citation indicators cannot be interpreted as directly comparable. The comparatively focused citation profile observed here is consistent with the narrower scope of AI-based infant pain detection, which intersects with AI, biomedical signal processing, neonatal medicine, and pain assessment. Importantly, the combination of rapid publication growth, a defined h-core, 579 unique author keywords, and more than 30,000 references indicates continued knowledge development and thematic diversification within the field. Taken together, these findings characterize AI-based infant pain detection as a rapidly developing, multidisciplinary, and internationally connected research domain that is progressively consolidating within neonatal healthcare informatics.

RQ2. How has the scientific output on AI-based infant pain detection evolved?

There is a clear pattern of increasing contributions in the temporal analysis, with limited initial contributions followed by a marked increase in both publication output and the number of contributors. Only two studies were published in the first year, 2010 (Figure 2). The number of papers published annually remained relatively low over the past decade, with the average number of papers per year remaining below 10. Nevertheless, these preliminary studies paved the way for methodological aspects of the field, especially in the fields of machine learning and pain assessment with images, which became the focus. In 2010, the number of citations received was exceptionally high (570), reflecting the importance of the publications as a foundation for further research.

Starting from 2020, there was a considerable boost in scientific output. There were nine papers published in 2020, eleven in 2021, sixteen in 2022, seventeen in 2023, twenty-five in 2024, and thirty-nine in 2025. This rising trend reflects growing research interest in neonatal pain assessment through advanced AI technologies, including deep learning, computer vision, and multimodal analytics. This increase is aligned with the trend of the AI sector in the health care industry, improvements in computational capacity, and the availability of more data for training the models. Citation indicators were used to assess the scholarly attention received by the publications. Publications from 2022 (497 citations) and 2023 (298 citations) had particularly strong influences, with some of the most impactful studies to help drive knowledge development in the field coming from these years (Table 2).

The distribution of publications also indicates a transition from exploratory research toward consolidation and expansion. Although the number of citations in recent years (2025–2026) is lower, this may be attributed to citation lag rather than necessarily reflecting reduced scholarly impact. The number of contributing authors and cited publications has steadily increased over time, indicating the expansion of the research base and community. Moreover, the rise in h-index and g-index values for individual years indicates an increasing number of highly cited publications within the corresponding annual cohorts compared with earlier years. As a whole, the temporal changes indicate that the research area of AI-based infant pain detection has shifted from a niche field to a more rapidly growing scientific field with rising publication numbers, number of participating researchers, and influence of the scientific community. The market is also poised for continued growth, given the potential of AI technologies in the clinical neonatal care setting.

To enable meaningful interpretation of the bibliometric metrics in Table 3, these indicators should be interpreted in relation to underlying mathematical objectives. The h-index measures baseline scientific output by identifying h papers with at least h citations, whereas the g-index gives weight to highly cited publications by calculating the highest number g of top papers that collectively accumulated at least $g^{2}$ citations. The m-quotient normalizes the h-index across the field’s 17-year timespan (h/y), where values above 1.0 denote an accelerated trajectory of scholarly growth. Crucially, the e-index complements the h-index by measuring the square root of “excess citations” accumulated by h-core papers above the baseline $h^{2}$ threshold, directly capturing lost citation impact. In domain-level citation analyses, e-index values under 5 indicate minimal excess citations (papers barely meeting the h-threshold), values between 5 and 10 denote moderate density, and values exceeding 10 reflect high excess impact driven by landmark studies. Within this corpus, peak annual e-index values such as 14.56 in 2020 and 18.84 in 2022 confirm that foundational papers in AI-driven infant pain detection have generated substantial, concentrated citation volume that significantly outpaces standard h-index projections.

RQ3. Which journals constitute the core publication sources in this research field?

The results of the publication source analysis indicate a relatively focused set of journals that serve as the primary publication outlets for AI-based infant pain detection research. Of these, *Biomedical Signal Processing and Control* and *Frontiers in Pediatrics* were the most productive sources, with eight articles published. These journals represent complementary areas of research, with the former focusing on signal processing and computational methodologies, and the latter focusing on clinical and pediatric applications. The high scores for these indicate that infant pain detection research is an interdisciplinary field that bridges technological innovation and neonatal healthcare practice (Figure 3).

While the number of publications is significant, the number of citations received by these publications provides additional insight into the scholarly attention associated with different journals. In terms of the number of citations per publication, *AI in Medicine* had the highest impact, with an average of 164.67 citations per publication across three publications (Table 4). Likewise, *Computer Methods and Programs in Biomedicine* had two publications with 90 citations, and the *IEEE Journal of Biomedical and Health Informatics* had five publications with 110 citations. The results indicate that there are many highly specialized journals, such as medical AI and biomedical and health informatics, that come with seminal studies which have a considerable impact on future studies. The high citation numbers of these journals suggest that their publications have contributed to the methodological development of the field and may have informed subsequent research.

The quality profile of top journals additionally indicates the scientific credibility of the research area. The core sources are mostly ranked in the first quartile (Q1) according to Scopus and Web of Science classifications: *Biomedical Signal Processing and Control*, *Frontiers in Pediatrics, AI in Medicine*, *IEEE Journal of Biomedical and Health Informatics*, *Neonatology*, and *Scientific Reports*. These journals also report established journal-level metrics, including CiteScore, SJR, and Journal Impact Factors. The high percentage of high-ranked journals suggests that studies related to AI-based infant pain detection are being published in well-established scholarly outlets with broad disciplinary visibility. These combined results indicate that the area has gained a considerable level of academic credibility and exposure, with a family of highly respected journals across biomedical engineering, AI, medical informatics, and paediatric healthcare fields.

RQ4. Which authors have made the most influential scholarly contributions to this field?

The analysis of influential contributions indicates that the literature on AI-based infant pain detection includes a relatively limited number of highly cited publications and authors. Nanni et al. [26], with 469 citations, was the most cited paper. While the research was more general and did not specifically focus on infant pain detection, it included methodological approaches relevant to AI-based pain recognition and may have provided methodological foundations for subsequent research. Likewise, the survey conducted by Werner et al. [27] (165 citations to date) has been an important reference for researchers who are interested in having a broad overview of automated pain assessment methodologies. The high number of citations of these publications indicates the significance of basic methodological research in the intellectual growth of the field (Table 5).

There are several significant authors directly involved in neonatal pain assessment technologies. One of the early machine learning-based methods for assessing pain intensity in neonates using digital imaging was developed by Gholami et al. [28] and has been cited 101 times, thus becoming an important research axis. Hariharan et al. [29] added to the field with their infant cry classification study, which garnered 88 citations and demonstrated the feasibility of the application of audio-based pain detection techniques. A recent study by Abbaskhah et al. [30] reported an increasing trend in deep learning-based cry classification systems, reflecting the recent advancements in the field of AI. Collectively, these studies illustrate the range of technological approaches investigated in the field, including facial image analysis, cry acoustics, physiological monitoring, and multimodal assessment.

The productivity of authors also reflects this difference between publications and scholarly impact. The authors who have published the most are Chakib Tadj. In contrast, authors like Alessandra Lumini have a lot more citation impact, with just two publications having been cited 500 times. This indicates that influential contributions do not necessarily have to be related to the number of published works, but to methodological importance and fundamental relevance. This also indicates an international character of knowledge production in this field, as there are influential researchers from all over the world, including Canada, Italy, Brazil, Türkiye, China, and the United States. In conclusion, the results indicate that methodological research, review papers, and innovative applications of machine learning techniques to neonatal healthcare problems have been the main drivers for the intellectual development of AI-based infant pain detection.

**Table 5:** Highly Cited Papers.

#	Authors	Title	Source	TC	C/Y	NTC
1	Nanni et al. (2010) [26]	Local binary patterns variants as texture descriptors for medical image analysis	*AI in Medicine*	469	27.59	1.65
2	Werner et al. (2022) [27]	Automatic Recognition Methods Supporting Pain Assessment: A Survey	*IEEE Transactions on Affective Computing*	165	33	5.31
3	Gholami et al. (2010) [28]	Relevance vector machine learning for neonate pain intensity assessment using digital imaging	*IEEE Transactions on Biomedical Engineering*	101	5.94	0.35
4	Chiera et al. (2020) [31]	Heart Rate Variability in the Perinatal Period: A Critical and Conceptual Review	*Frontiers in Neuroscience*	92	13.14	3
5	Hariharan et al. (2018) [29]	Improved binary dragonfly optimization algorithm and wavelet packet-based non-linear features for infant cry classification	*Computer Methods and Programs in Biomedicine*	88	9.78	1.21
6	Motrico et al. (2021) [32]	Impact of the COVID-19 pandemic on perinatal mental health (Riseup-PPD-COVID-19): protocol for an international prospective cohort study	*BMC Public Health*	61	10.17	3.23
7	Abbaskhah et al. (2023) [30]	Infant cry classification by MFCC feature extraction with MLP and CNN structures	*Biomedical Signal Processing and Control*	60	15	3.42
8	Aloraynan et al. (2022) [33]	A Single Wavelength Mid-Infrared Photoacoustic Spectroscopy for Noninvasive Glucose Detection Using Machine Learning	*Biosensors*	58	11.6	1.87
9	Khanmohammadi & Chou (2018) [34]	Adaptive Seizure Onset Detection Framework Using a Hybrid PCA-CSP Approach	*IEEE Journal of Biomedical and Health Informatics*	58	6.44	0.79
10	Neumann et al. (2017) [35]	Quality of compounded hydrocortisone capsules used in the treatment of children	*European Journal of Endocrinology*	50	5	1.02

Note: TC = Total Citations; C/Y = Citations per Year; NTC = Normalised Total Citations.

RQ5. What is the relationship between author productivity and citation impact in this research domain?

There seems to be a non-linear correlation between productivity and citation performance in AI-based infant pain detection research. Highly cited authors are not necessarily the most productive authors. For instance, Chakib Tadj had the third-highest number of publications (3) but received only 16 citations (Figure 4). On the other hand, Alessandra Lumini published two papers, but received an extraordinary 500 citations, giving her an average of 250 citations per publication. The difference indicates that the importance and novelty of the research contributions are more important in determining the impact of citations than the number of publications per se.

This trend is also reflected in other prominent researchers. Some authors with relatively few publications, such as Lucas Pereira Carlini and Tatiany Marcondes Heiderich, each published two papers that received a total of 39 citations, resulting in relatively high average citation counts compared with those of other authors. In a similar vein, Peter R. Mouton received 18 citations from two publications, although he only had one cited paper. These results indicate that high-impact publications can significantly help boost an author’s scholarly impact despite limited publications. Therefore, single productivity indicators might not be sufficient to measure academic impact in this new research field (Table 6).

The citation indicators further indicate that influential research may emerge from studies addressing methodological innovations or clinical applications. The researchers who had a higher h-index, g-index and citations per publication tended to have contributed studies addressing important technical challenges like pain recognition algorithms, multimodal analysis, or neonatal-specific AI applications. Therefore, productivity is related to impact in terms of quality and not quantity. For a growing and developing field like AI, the number of citations driven by impact would likely be more concentrated on those researchers who create widely used data sets, methods, and clinically validated AI systems. Overall, the findings indicate that the number of publications in the field of AI-based Infant Pain Detection does not necessarily signify its scholarly impact; rather, the impact of the publications is the most critical, which is characteristic of a highly specialized and innovative field.

RQ6. What are the global patterns of scientific contributions, and which geographical regions remain underrepresented?

The spatial distribution of scientific production reveals a high concentration of research output among a small number of countries. The United States contributed 34 publications and 1267 citations, exceeding other countries in both publication output and citation counts (Figure 5). China had the second-highest number of publications, with 29, followed by India with 20 publications. Together, these three countries combined produce a significant amount of research output, demonstrating the leading role of the main scientific and technological economies in research on infant pain detection using AI.

In addition to the number of publications, differences in citation performance across countries citation performance across, as indicated by the citation measures. Italy had the highest average citation rate (71 citations per publication), with only 9 publications that have received a total of 639 citations. Likewise, Germany, Brazil, France, and Spain had relatively high citation rates, compared to their publication numbers (Table 7). The results indicate that some European countries received substantial scholarly attention despite having lower publication output. The high citation rates seen in these countries could be related to methodological studies of high quality and research collaboration, as well as to the application of AI technologies in the clinical environment in neonatal health care, and to their validation in clinical settings.

Significant geographical disparities still exist, although the number of countries represented in literature is growing. Many regions are underrepresented, and research contributions are heavily concentrated in North America, Europe, and parts of Asia. The dataset provides limited representation of African countries, as Morocco is the only country identified as contributing to the data. Similarly, the influence from Latin America, the Middle East, and Southeast Asia is relatively limited. This distribution may be associated with differences in research funding, technology infrastructure, clinical data availability, and access to AI expertise. The absence of developing regions is a concern, as it suggests that existing AI models, primarily developed for high-income countries, might not be broadly applicable. Future research should also aim to broaden geographical representation and strengthen international collaborations to assess the applicability of AI-based infant pain detection systems in various healthcare settings and population groups.

RQ7. What are the dominant research themes and emerging knowledge structures in AI-based infant pain detection?

The results indicate that the keywords “AI” and “infant pain detection” are co-occurring, suggesting that the research on AI-based infant pain detection primarily relies on machine learning. The term “machine learning” was used most frequently (50) and had a high network connectivity (Figure 6). This finding indicates that machine learning techniques remain the primary approach to analyzing infant pain signals. This strong algorithmic focus is confirmed by tight keyword clusters connecting “machine learning” to related technical terms, including “deep learning,” “artificial intelligence,” “classification”, and “predictive model.” These are considered to be the fundamental set of words in knowledge when it comes to algorithm development and automated pain recognition.

The second group of themes concerns the clinical applications and contexts of neonatal healthcare. The terms are “neonatal intensive care unit,” “newborn,” “infant,” and “pain assessment,” which represent the real-world context in which the AI technologies are applied. The high frequency of these terms suggests a growing interest among researchers in translating advances in computational science into clinical applications that can support healthcare professionals in the neonatal care setting. In summary, AI’s role in the NICU is indicative of a broader shift in healthcare towards real-time monitoring, objective evaluation, and decision support systems, all of which aim to improve pain management in fragile patient groups.

A number of new knowledge structures have also been discovered that are influencing the future research directions. The average publication year of terms like “deep learning,” “CNN,” and “predictive model” is relatively recent, reflecting the increasing number of recent publications addressing these topics. In contrast, “support vector machine” and traditional machine learning techniques have earlier publication dates, indicating a shift to more advanced deep learning architectures. As the field progresses beyond classification of pain, we are seeing a rise in the use of neural networks, multimodal analytics and predictive modelling in the development of continuous and complete pain monitoring systems. In total, the overall conceptual structure indicates that the research on machine learning has developed from basic machine learning research to more complex AI-based systems with clinical relevance, multi-modality data processing and predictive health care functions (Table 8).

The quantitative transition from early single-modality models to advanced monitoring architectures is reflected in the average publication years of key thematic terms in Table 8. Classical machine learning approaches and binary algorithms, such as SVM, have an earlier average publication year (2019.0, average citations =114.50). In contrast, deep learning frameworks (2024.5), CNNs (2023.8), and continuous predictive models (2024.0) represent significantly newer intellectual nodes. This empirical progression in keyword recency proves that research focus has systematically transitioned from static, single-source binary classification toward real-time, multimodal deep learning architectures capable of continuous clinical monitoring in neonatal care settings.

RQ8. What are the patterns of international research collaboration in AI-based infant pain detection, as revealed through country co-authorship networks?

The country co-authorship network indicates that international co-authorship is a prominent feature of research on infant pain detection using AI. The high rate of international co-authorship in the overall dataset is evident in the network structure, where multiple countries are connected through their collaborative publications. The United States is the most central, with the highest total link strength and 33 documents in collaboration. This centrality indicates that the United States has extensive collaborative connections with researchers from various countries, facilitating the exchange of expertise, data, and technological resources (Figure 7).

There are several distinct collaboration clusters that can be discerned from the network. China forms a highly productive research cluster but has relatively fewer international connections than the United States. India and Türkiye form another partnership group, which is indicative of the increase in research activities in emerging scientific economies. International collaborations are also evident among European countries, including Germany, the United Kingdom, Italy, and Canada. The clusters suggest that the organization of knowledge production is based on regional and institutional collaborations, which are key to the process of methodological innovation and clinical validation (Table 9).

The collaboration network also indicates opportunities to strengthen the integration of global research. Some countries, like Saudi Arabia, South Korea, and Australia, are involved in collaborative activities, but exhibit relatively weak network connectivity. Moreover, many developing regions have limited representation in the co-authorship map, indicating uneven participation in international research networks. Scaling up partnerships to less-represented areas could help diversify datasets, increase model generalizability and help ensure equitable technological development. The collaborative efforts of international partners will likely continue to be important in the future to address methodological challenges, to validate algorithms across populations, and to facilitate and speed up translation from research results to clinical use in infant pain detection using AI. Overall, the co-authorship network as a whole demonstrates a global network but with an uneven distribution of research, with a few countries having a large number of connections and being in the center of the network, thus contributing to scientific progress in this area.

## 6. Discussion

The rapid expansion of research on AI-based infant pain detection reflects a broader transition in neonatal care from intermittent and observer-dependent pain assessment toward automated, continuous, and potentially multimodal monitoring. The significance of this development extends beyond the increase in publication activity. Infant pain assessment remains particularly challenging because neonates cannot provide verbal self-report. At the same time, behavioral and physiological indicators may be subtle, heterogeneous, and influenced by factors other than pain. In this context, the growing integration of AI, computer vision, biomedical signal processing, and clinical assessment suggests that the field is increasingly addressing not only whether pain can be detected automatically, but also how objective and continuous indicators could complement conventional clinical assessment. Nevertheless, the bibliometric evidence should be interpreted as an indication of increasing scientific attention and knowledge development rather than as evidence that AI-based systems have already achieved clinical effectiveness or routine bedside readiness. The geographical concentration of research provides an important insight into the structural development of the field. The prominence of the United States and several European and Asian countries may plausibly reflect differences in research funding, advanced computational infrastructure, established neonatal research networks, and access to specialized clinical datasets. However, these factors were not directly measured in the present bibliometric analysis and therefore should be regarded as plausible explanations rather than demonstrated causal determinants. More importantly, geographical concentration has important methodological implications for AI development. Models trained predominantly using data from particular institutions, populations, equipment, or clinical environments may encounter distributional differences when deployed in other settings. Variations in neonatal demographics, clinical practices, camera and sensor characteristics, environmental conditions, and pain-assessment protocols can potentially affect model performance. Thus, the geographical distribution observed in this review is not merely a matter of research productivity; it raises a broader question concerning whether current AI systems are being developed and evaluated across sufficiently diverse neonatal populations and care environments.

The exceptionally high proportion of international co-authorship provides a complementary perspective. The observed collaboration pattern may reflect the inherently multidisciplinary and data-intensive nature of infant pain research. High-quality AI development requires expertise spanning neonatal medicine, nursing, biomedical engineering, computer science, signal processing, and health informatics. At the same time, the ethical collection of sufficiently large infant datasets can be challenging for individual research centers. Multicenter and international collaboration can therefore facilitate access to larger and more heterogeneous datasets and enable researchers to assess whether algorithms maintain reliable performance across different populations and clinical settings. This is particularly important for infant pain detection because model generalizability cannot be adequately established through repeated evaluation within a single institution. Future collaborative studies should consequently move beyond co-authorship and establish shared protocols for data acquisition, annotation, preprocessing, outcome definition, and external validation. Such collaboration would provide a stronger basis for determining whether apparently high-performing models retain their performance when transferred across hospitals, countries, and clinical workflows. The thematic evolution of the literature also highlights an important translational gap. The increasing attention to deep learning, automated recognition, and multimodal approaches indicates substantial progress in algorithmic development; however, technical performance alone does not establish clinical utility. A model that accurately distinguishes pain from non-pain conditions under controlled experimental conditions may still perform differently in real neonatal intensive care environments, where infants experience multiple concurrent stimuli and physiological states. Accordingly, future research should place greater emphasis on external and prospective validation rather than relying predominantly on internally derived performance estimates. Studies should report performance across independent institutions and clinically relevant subgroups, together with calibration, robustness, missing-data behavior, and failure modes. These considerations are particularly important when models are intended to support clinical decision-making, where false-positive and false-negative assessments may have different consequences.

Another important gap concerns the representation of pain as a dynamic clinical phenomenon. Much of the development of automated recognition systems has centered on classification tasks. In contrast, clinically meaningful pain assessment may require continuous or repeated estimation of pain intensity and trajectory. Future systems could therefore progress from static “pain versus no pain” classification toward interpretable monitoring of changes in pain-related behavioral and physiological patterns over time. Such development should also explicitly address potential confounding states, including hunger, fatigue, agitation, handling, and other forms of neonatal distress that may produce overlapping behavioral or physiological responses. Multimodal architectures integrating facial behavior, vocalization or crying, and physiological signals may offer one pathway for addressing this complexity. However, their value should be demonstrated through rigorous cross-modal validation rather than assumed from increased technical complexity. The findings also have implications for equity and implementation. The concentration of research in a relatively limited number of countries creates a risk that datasets and algorithms may reflect the characteristics of well-resourced healthcare environments more strongly than those of resource-constrained settings. Expanding research participation to underrepresented regions is therefore important not simply to increase geographical diversity, but to test whether AI systems remain reliable under different clinical, technological, and demographic conditions. Researchers should consider multicenter validation involving diverse healthcare settings, including low- and middle-income environments, and should report the contextual characteristics necessary to understand where a model can and cannot be reliably deployed. This would shift the research agenda from developing increasingly sophisticated algorithms toward establishing where, for whom, and under what conditions those algorithms provide dependable clinical support.

Based on these findings, several concrete priorities emerge for the next stage of research. First, collaborative networks should develop ethically governed, multicenter benchmark datasets containing sufficiently diverse infant populations and standardized pain annotations. Second, future studies should prioritize independent external and prospective validation across hospitals rather than reporting performance exclusively from the development cohort. Third, multimodal models should be evaluated against unimodal approaches to determine whether additional data streams provide meaningful clinical benefit rather than only higher computational complexity. Fourth, explainability, robustness, calibration, and clinically relevant failure analysis should become integral components of model evaluation. Finally, research should increasingly include underrepresented populations and healthcare settings to assess generalizability and reduce the risk of context-dependent algorithmic bias. Together, these priorities would help bridge the current gap between proof-of-concept AI research and clinically credible infant pain-monitoring systems. The central challenge for the field is therefore no longer solely whether AI can detect indicators of infant pain, but whether such systems can provide reliable, interpretable, generalizable, and clinically useful information across diverse neonatal care environments.

## 7. Limitations

Although this study provides a comprehensive quantitative mapping of research on AI-based infant pain detection, several methodological limitations should be acknowledged. First, the literature retrieval was restricted exclusively to the Scopus database. Although Scopus provides extensive coverage of high-impact biomedical, healthcare, and computer science publications, relevant studies indexed only in other databases, such as Web of Science, IEEE Xplore, or PubMed, may not have been captured. Second, the search was limited to English-language publications, potentially excluding relevant non-English studies and regional clinical evidence. Third, the corpus included exclusively final peer-reviewed journal articles, while conference proceedings, book chapters, and preprints were excluded. While this criterion ensured high consistency in publication quality, peer-review standards, and bibliographic metadata, it may underrepresent the most recent methodological developments. In AI, computer vision, and machine learning, novel architectures and feature extraction techniques are frequently reported first in high-impact engineering conference proceedings (such as IEEE or ACM events). Consequently, omitting conference literature represents a trade-off between metadata completeness and catching ultra-recent algorithmic prototypes. Future bibliometric mapping studies should consider expanding their criteria to include major peer-reviewed conference proceedings to capture emerging technical prototypes alongside validated clinical journal outputs. In addition, the 2026 dataset represents only a partial six-month publication window, as the data were retrieved in June 2026, and therefore should not be interpreted as representing the complete annual research output.

Beyond the methodological limitations of the bibliometric analysis, limitations within the existing primary literature should also be considered. Many existing studies rely on relatively small and isolated datasets collected under controlled experimental conditions, which may limit the generalizability and clinical translation of AI models across different hospitals, patient populations, and care environments. Model performance may also be affected by practical challenges such as facial occlusion, variations in illumination, changes in infant positioning, and the presence of multiple sources of distress that may produce similar behavioral or acoustic signals. These challenges highlight the need for stronger external validation using independent datasets, multi-center collaborations, standardized data collection protocols, and clinically representative environments. Future bibliometric and systematic investigations should therefore consider integrating multiple databases, expanding language coverage, incorporating relevant conference literature, and monitoring the field using more comprehensive and continuously updated datasets.

## 8. Conclusions

This bibliometric analysis provides a comprehensive overview of the development, intellectual structure, and emerging directions of AI research for pain detection in nonverbal infants between 2010 and 2026. The findings demonstrate that this research area is dynamic and rapidly consolidating, with an estimated annual growth rate of approximately 29% and a marked increase in publication activity after 2020. The literature is relatively recent, with an average document age of less than three years and a high level of author collaboration, reflecting the interdisciplinary nature of the field. Thematic analysis further indicates a clear transition from conventional approaches based on individual modalities, such as facial expressions or infant crying, towards more sophisticated approaches integrating multiple sources of information, including video, audio, and physiological signals. At the same time, developments in deep learning, computer vision, and advanced signal processing are increasingly shifting the field from experimental algorithm development towards more clinically oriented and potentially real-time applications.

The study also highlights the importance of multimodal and explainable AI as key directions for the future development of automated infant pain monitoring. Rather than relying solely on binary pain classification, future systems should increasingly support continuous assessment of behavioral and physiological changes while providing interpretable outputs that can assist healthcare professionals in clinical decision-making. Such developments could contribute to more objective and timely recognition of pain, particularly in neonatal and intensive care environments where infants are unable to communicate their discomfort verbally. Nevertheless, successful clinical translation will require more than improvements in algorithmic performance. Robust external validation, diverse multi-center datasets, standardized evaluation protocols, ethical considerations, and close collaboration between AI researchers, biomedical engineers, and pediatric healthcare professionals are essential. Overall, the findings position AI-based infant pain detection as an emerging multidisciplinary research field in which technological innovation must be closely aligned with clinical requirements, reliability, interpretability, and patient safety.

## Figures and Tables

**Figure 1:**
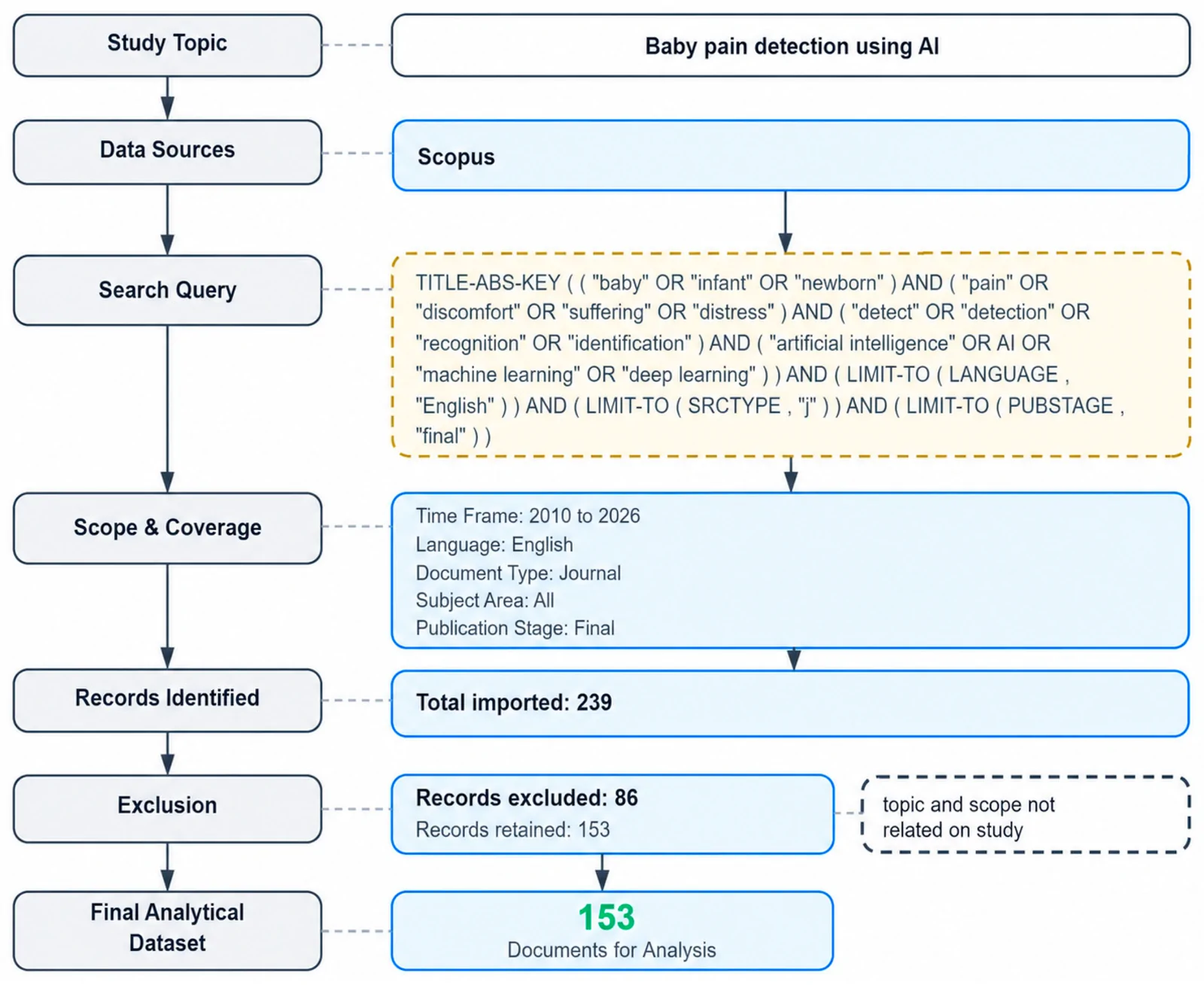
Bibliometric Study Flow Diagram.

**Figure 2:**
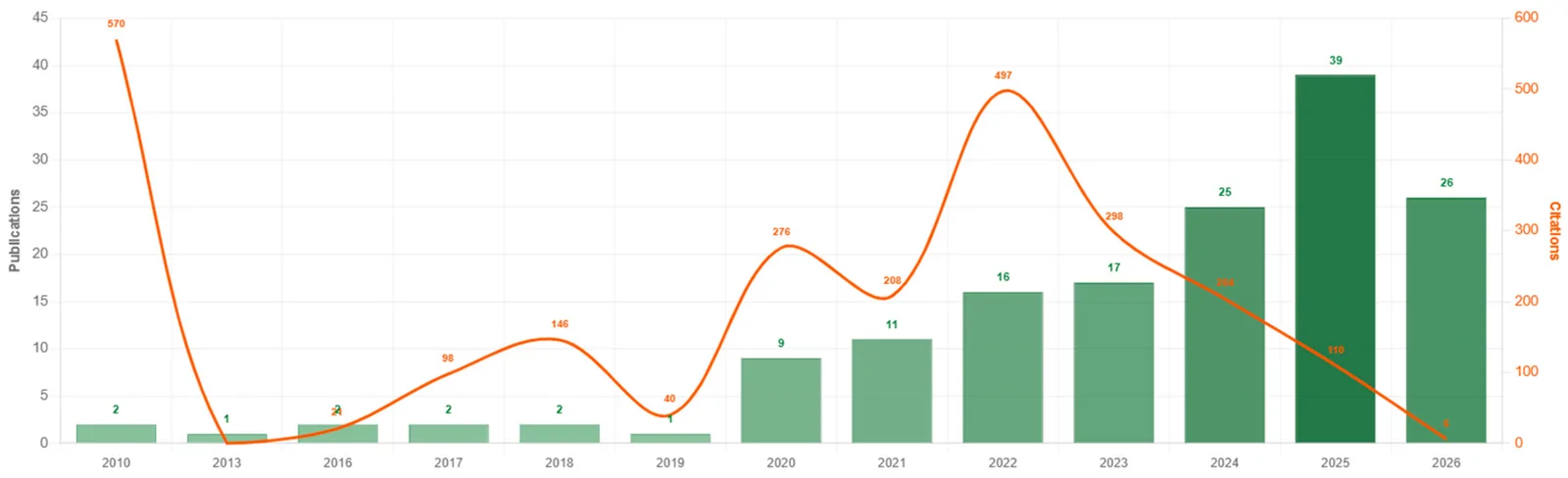
Annual Publications and Citations.

**Figure 3:**
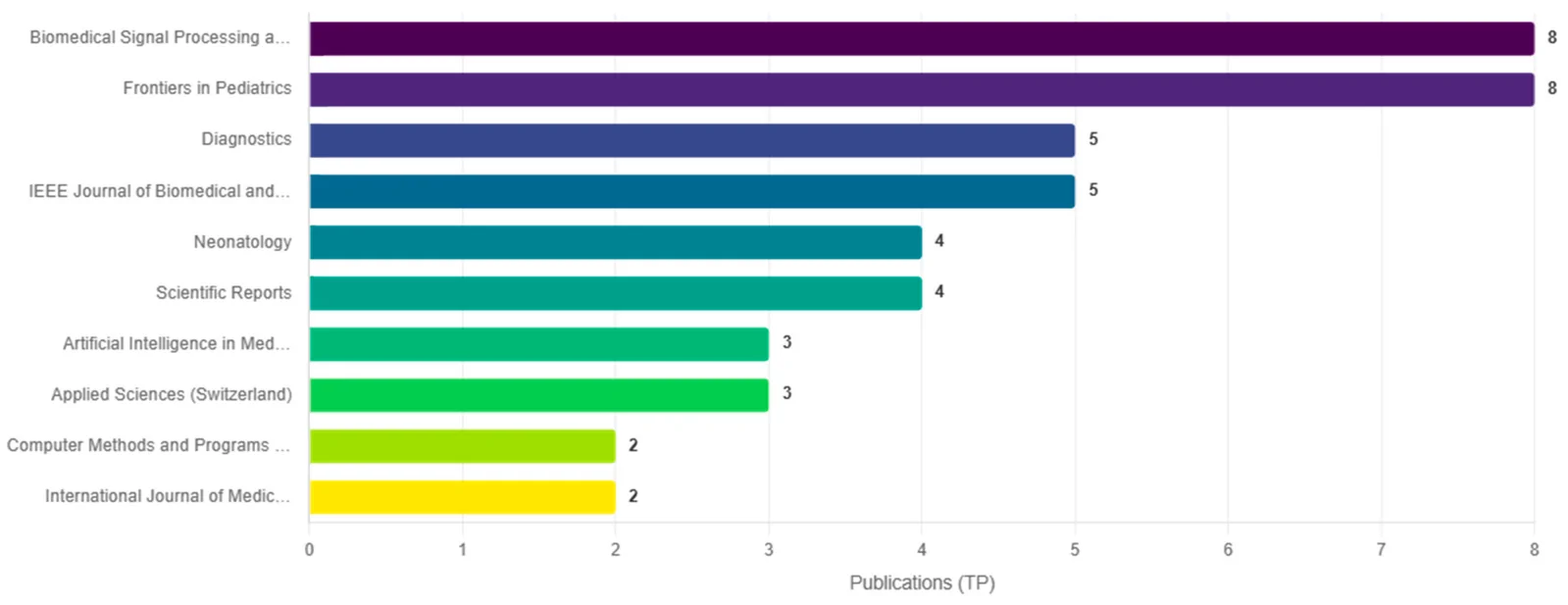
Top Sources.

**Figure 4:**
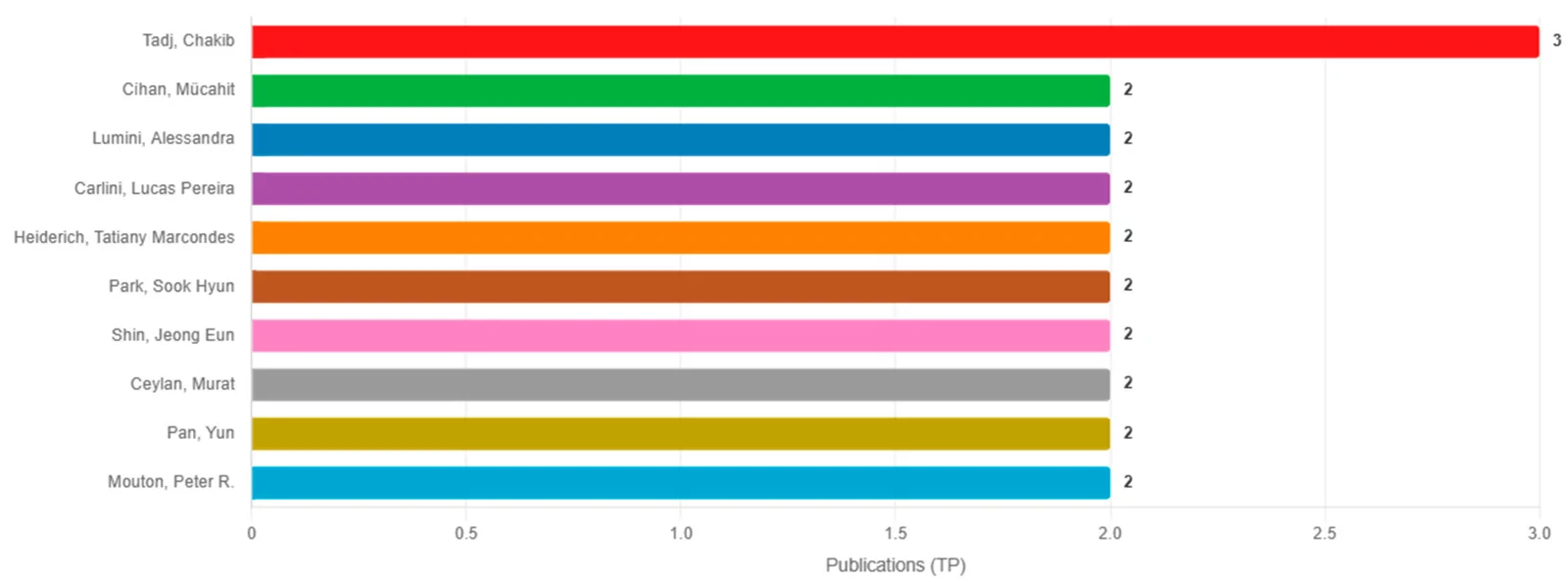
Top Authors.

**Figure 5:**
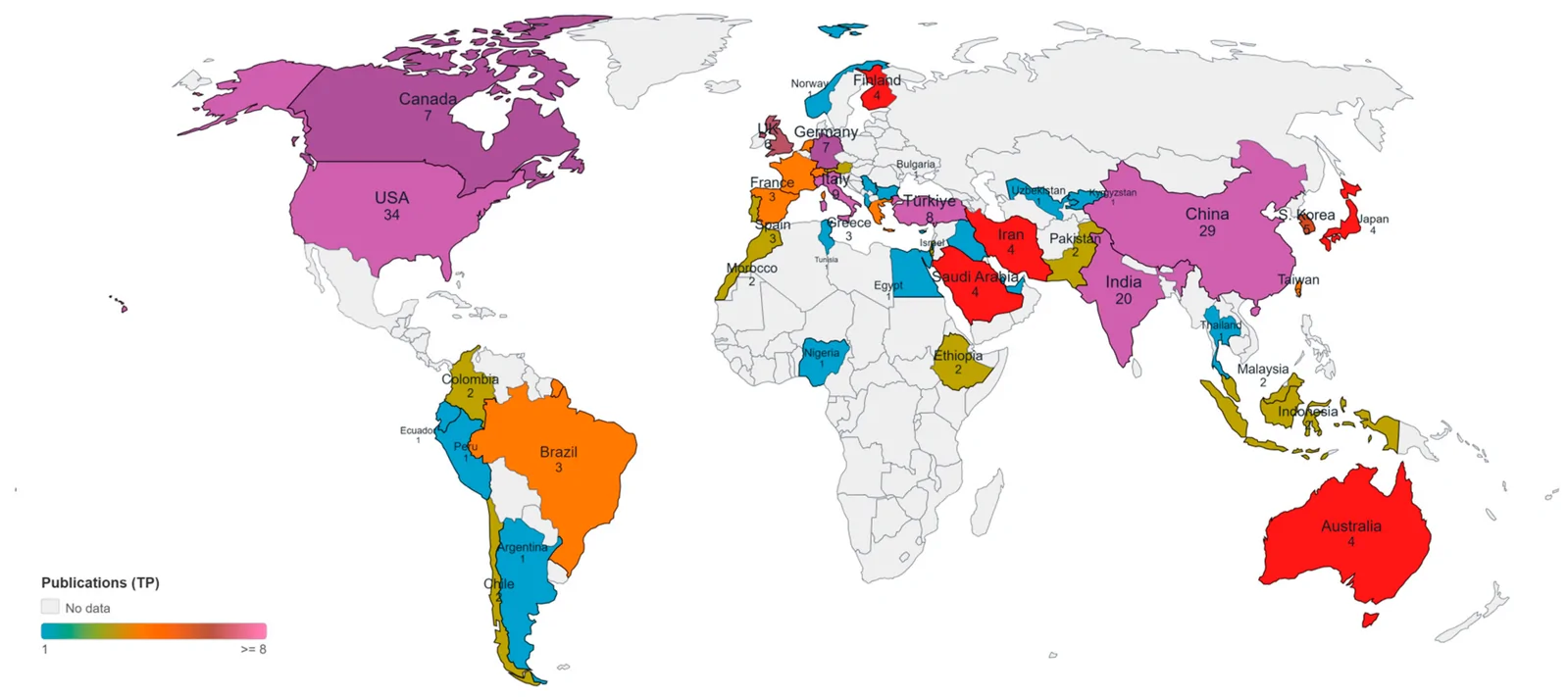
World Map.

**Figure 6:**
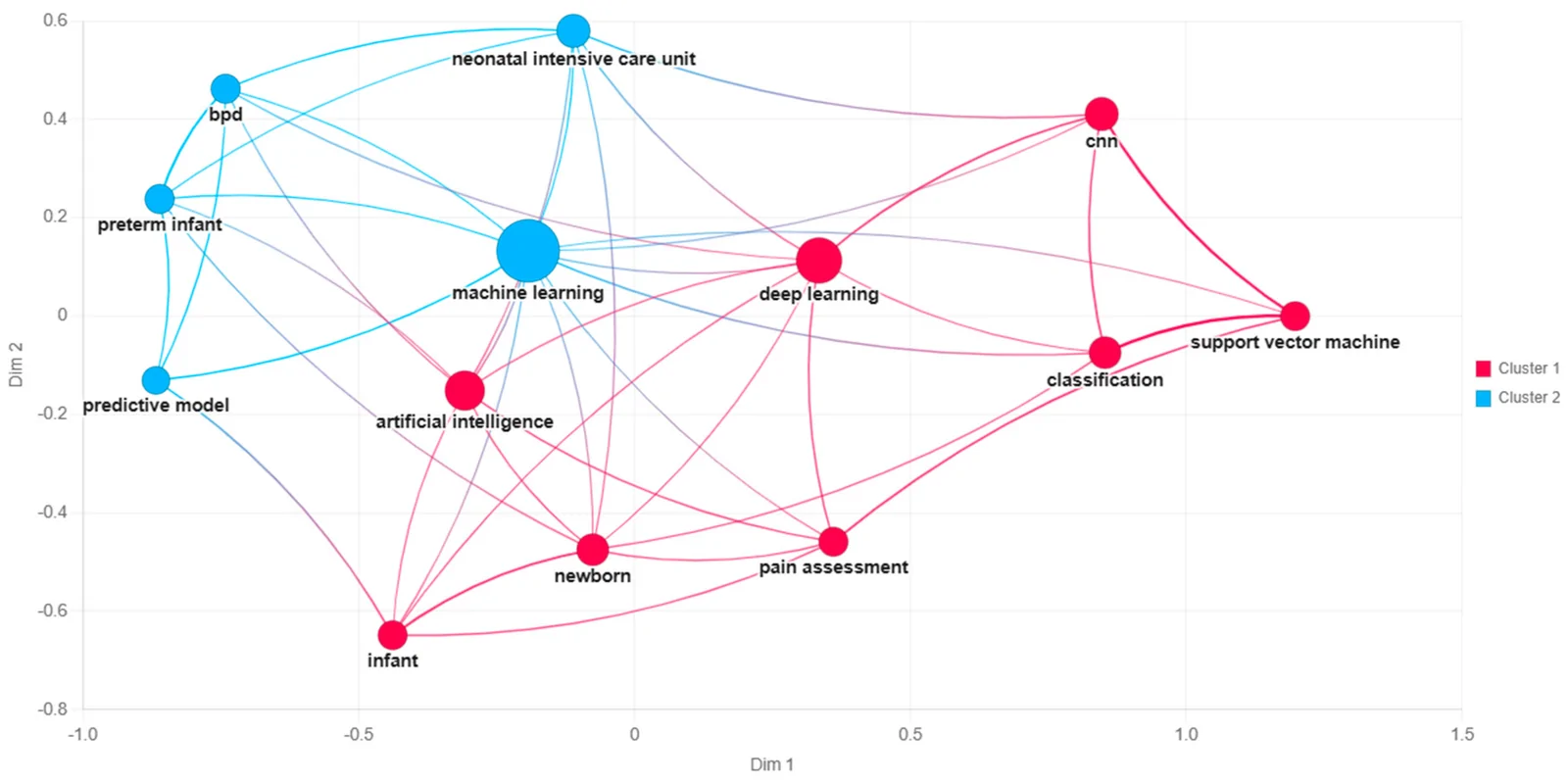
Network Visualization of Author Keywords.

**Figure 7:**
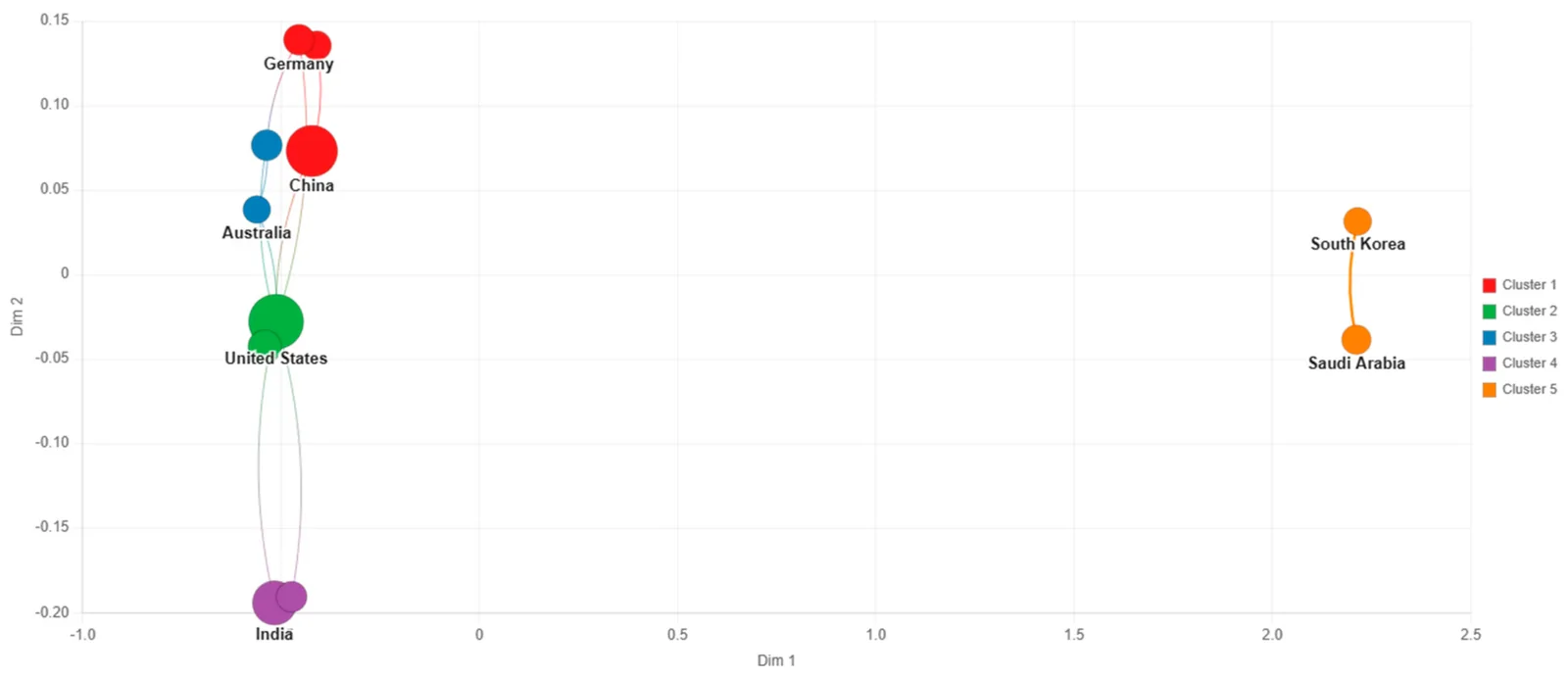
Network Map of Countries.

**Table 1:** The Search String.

Scopus	TITLE-ABS-KEY ((“baby” OR “infant” OR “newborn”) AND (“pain” OR “discomfort” OR “suffering” OR “ distress”) AND (“detect” OR “detection” OR “recognition” OR “identification”) AND (“artificial intelligence” OR AI OR “machine learning” OR “deep learning”)) AND (LIMIT-TO (LANGUAGE, “English”)) AND (LIMIT-TO (SRCTYPE, “j”)) AND (LIMIT-TO (PUBSTAGE, “final”)). Date of Access: June 2026

**Table 2:** Bibliometric Summary.

Indicator	Value
Timespan	2010:2026
Total Publications (TP)	153
Citable Year Span	17 years
Annual Growth Rate	29.06%
Document Average Age	2.74 years
Total Citations (TC)	2474
Average Citations per Document	16.17
Number of Cited Papers (NCP)	119
Average Citations per Cited Paper	20.79
Average Citations per Year	145.53
Total Contributing Authors	930
Total Unique Authors	854
Total Institutions	411
Total Countries	55
Single-Authored Documents	4
Average Authors per Paper	6.08
International Co-authorship Rate	88.24%
Unique Author Keywords	579
Total References	30,138
h-index	26
g-index	46
m-index	1.529
Citation Sum within h-Core	1759

**Table 3:** Yearly Bibliometric Statistics.

Year	TP	NCA	NCP	TC	C/P	C/CP	h	g	m	e
2026	26	151	4	6	0.23	1.5	1	2	0.059	1.41
2025	39	250	32	110	2.82	3.44	6	8	0.375	4.69
2024	25	139	23	204	8.16	8.87	9	13	0.6	8.19
2023	17	114	17	298	17.53	17.53	10	17	0.714	12.33
2022	16	83	16	497	31.06	31.06	10	16	0.769	18.84
2021	11	83	11	208	18.91	18.91	8	11	0.667	11.4
2020	9	48	8	276	30.67	34.5	8	9	0.727	14.56
2019	1	7	1	40	40	40	1	1	0.1	6.24
2018	2	9	2	146	73	73	2	2	0.222	11.92
2017	2	10	2	98	49	49	2	2	0.25	9.7
2016	2	7	1	21	10.5	21	1	2	0.143	4.47
2013	1	10	0	0	0	0	0	0	0	0
2010	2	6	2	570	285	285	2	2	2	23.79

Note: TP = Total Publications; NCA = Number of Contributing Authors; NCP = Number of Cited Publications; TC = Total Citations; C/P = Citations per Publication; C/CP = Citations per Cited Publication; h = h-index; g = g-index; m = m-Quotient; e = e-index.

**Table 4:** Source Statistics.

**Source Title**	Publisher	TP	TC	NCA	NCP	C/P	C/CP	h	g	m	e	CiteScore	SJR	SNIP	Q (Scopus)	JIF	Q (WoS)	ABDC	AJG
*Biomedical Signal Processing and Control*	Elsevier Ltd.	8	163	27	6	20.375	27.167	5	8	0.714	11.62	11.5	1.229	1.65	Q1	4.9	Q2	nan	nan
*Frontiers in Pediatrics*	Frontiers Media SA	8	69	44	8	8.625	8.625	5	8	1	6.24	4.3	0.738	0.941	Q1	2.0	Q2	nan	nan
*Diagnostics*	Multidisciplinary Digital Publishing Institute (MDPI)	5	19	30	4	3.8	4.75	2	4	0.5	3.32	5.9	0.773	1.08	Q2	3.3	Q1	nan	nan
*IEEE Journal of Biomedical and Health Informatics*	Institute of Electrical and Electronics Engineers Inc.	5	110	26	5	22	22	5	5	0.714	9.22	13.5	1.649	1.975	Q1	6.8	Q1	nan	nan
*Neonatology*	S. Karger AG	4	22	33	4	5.5	5.5	2	4	0.5	3.87	5.5	0.901	1.241	Q1	3.0	Q1	nan	nan
*Scientific Reports*	Nature Research	4	26	30	4	6.5	6.5	2	4	1	4.36	6.7	0.874	1.213	Q1	3.9	Q1	nan	nan
*Artificial Intelligence in Medicine*	Elsevier B.V.	3	494	17	3	164.667	164.667	3	3	0.188	22.02	12.2	1.396	1.93	Q1	6.2	Q1	nan	nan
*Applied Sciences* (Switzerland)	MDPI	3	45	17	2	15	22.5	2	3	0.286	6.4	5.5	0.521	0.956	Q1	2.5	Q2	nan	nan
*Computer Methods and Programs in Biomedicine*	Elsevier Ireland Ltd.	2	90	18	2	45	45	2	2	0.25	9.27	11.1	1.13	1.627	Q1	4.8	Q1	nan	nan
*International Journal of Medical Informatics*	Elsevier Ireland Ltd.	2	3	10	1	1.5	3	1	1	0.5	1.41	7.9	1.189	1.547	Q1	4.1	Q1	A	nan

Note: TP = Total Publications; TC = Total Citations; NCA = Number of Contributing Authors; NCP = Number of Cited Publications; C/P = Citations per Publication; C/CP = Citations per Cited Publication; h = h-index; g = g-index; m = m-Quotient; e = e-index; SJR = SCImago Journal Rank; SNIP = Source-Normalised Impact per Paper; JIF = Journal Impact Factor; ABDC = Australian Business Deans Council Rating; AJG = Academic Journal Guide.

**Table 6:** Author Statistics.

Author	Affiliation	Country	TP	NCP	TC	C/P	C/CP	h	g	m	e
Tadj, Chakib	Université Du Québec	Canada	3	3	16	5.33	5.33	1	3	0.25	3.61
Cihan, Mücahit	Konya Technical University	Türkiye	2	2	9	4.50	4.50	2	2	2.00	2.24
Lumini, Alessandra	University of Bologna	Italy	2	2	500	250.00	250.00	2	2	0.14	22.27
Carlini, Lucas Pereira	University College Fei	Brazil	2	2	39	19.50	19.50	2	2	1.00	5.92
Heiderich, Tatiany Marcondes	University College Fei	Brazil	2	2	39	19.50	19.50	2	2	1.00	5.92
Park, Sook Hyun	Invisionlab Inc.	South Korea	2	1	11	5.50	11.00	1	2	0.25	3.16
Shin, Jeong Eun	Yonsei University College of Medicine	South Korea	2	1	11	5.50	11.00	1	2	0.25	3.16
Ceylan, Murat	Selçuk University	Türkiye	2	2	9	4.50	4.50	2	2	2.00	2.24
Pan, Yun	Zhejiang University	China	2	2	14	7.00	7.00	2	2	1.00	3.16
Mouton, Peter R.	University of South Florida	United States	2	1	18	9.00	18.00	1	2	0.33	4.12

Note: TP = Total Publications; NCP = Number of Cited Publications; TC = Total Citations; C/P = Citations per Publication; C/CP = Citations per Cited Publication; h = h-index; g = g-index; m = m-Quotient; e = e-index.

**Table 7:** Country Statistics.

Country	TP	NCA	NCP	TC	C/P	C/CP	h	g	m	e
United States	34	192	31	1267	37.26	40.87	16	34	0.941	30.05
China	29	223	20	200	6.9	10	8	13	1.143	9.8
India	20	77	14	209	10.45	14.93	7	14	0.636	12
Italy	9	57	6	639	71	106.5	6	9	0.353	24.56
Türkiye	8	57	7	199	24.88	28.43	5	8	0.455	13.08
Canada	7	21	7	140	20	20	5	7	0.556	10.63
Germany	7	49	5	261	37.29	52.2	4	7	0.4	15.62
United Kingdom	6	69	5	121	20.17	24.2	3	6	0.3	10.34
South Korea	5	34	2	32	6.4	16	2	5	0.5	5.29
Australia	4	17	4	46	11.5	11.5	2	4	0.4	6.32
Finland	4	32	3	15	3.75	5	2	3	0.4	3.16
Saudi Arabia	4	27	2	10	2.5	5	1	3	0.2	2.83
Japan	4	23	4	16	4	4	3	4	1	2.45
Iran	4	24	4	89	22.25	22.25	3	4	0.5	8.83
France	3	40	3	81	27	27	3	3	0.6	8.49
Brazil	3	40	3	100	33.33	33.33	3	3	0.75	9.54
Greece	3	41	2	69	23	34.5	2	3	0.333	8.06
Spain	3	40	2	80	26.67	40	2	3	0.286	8.72
Switzerland	3	21	3	37	12.33	12.33	2	3	0.4	5.57
Taiwan	3	16	3	55	18.33	18.33	2	3	0.667	7
Netherlands	3	9	3	37	12.33	12.33	3	3	1	5.29
Portugal	2	35	2	63	31.5	31.5	2	2	0.4	7.68
Pakistan	2	21	1	9	4.5	9	1	2	0.2	2.83
Israel	2	30	1	61	30.5	61	1	2	0.2	7.75
Morocco	2	17	1	6	3	6	1	2	1	2.24

Note: TP = Total Publications; NCA = Number of Contributing Authors; NCP = Number of Cited Publications; TC = Total Citations; C/P = Citations per Publication; C/CP = Citations per Cited Publication; h = h-index; g = g-index; m = m-Quotient; e = e-index.

**Table 8:** Keyword Co-Occurrence Metrics, Cluster Associations, and Temporal Recency (2010–2026).

#	Term	Cluster	Occurrences	Links	Total Link Strength	Relevance	Avg. Pub. Year	Avg. Citations	Avg. Norm. Citations	X	Y
1	machine learning	2	50	12	43.00	50.000	2023.6	9.60	0.67	−0.193	0.133
2	deep learning	1	22	9	17.00	22.000	2024.5	10.73	1.03	0.335	0.113
3	artificial intelligence	1	15	8	23.00	15.000	2024.3	8.53	0.51	−0.307	−0.151
4	cnn	1	9	5	6.00	9.000	2023.8	11.89	2.91	0.847	0.411
5	neonatal intensive care unit	2	9	7	13.00	9.000	2024.0	14.67	0.89	−0.111	0.580
6	classification	1	8	5	10.00	8.000	2023.4	13.63	1.50	0.853	−0.074
7	newborn	1	8	8	11.00	8.000	2022.6	22.13	0.95	−0.075	−0.475
8	preterm infant	2	6	6	11.00	6.000	2023.8	19.83	1.25	−0.861	0.238
9	bpd	2	6	6	13.00	6.000	2024.0	9.67	1.18	−0.741	0.462
10	support vector machine	1	6	4	5.00	6.000	2019.0	114.50	2.49	1.198	0.000
11	infant	1	6	6	8.00	6.000	2024.7	9.33	0.88	−0.438	−0.649
12	pain assessment	1	6	6	10.00	6.000	2020.7	59.33	1.62	0.361	−0.459
13	predictive model	2	5	4	8.00	5.000	2024.0	11.20	1.16	−0.867	−0.131

**Table 9:** Statistics by Country.

#	Country	Cluster	Documents	Links	Total Link Strength	Relevance	Avg. Pub. Year	Avg. Citations	Avg. Norm. Citations	X	Y
1	United States	2	33	7	13.00	33.000	2021.8	34.24	1.09	−0.512	−0.028
2	China	1	28	2	4.00	28.000	2023.8	8.96	0.59	−0.422	0.073
3	India	4	19	2	2.00	19.000	2023.4	11.00	1.69	−0.516	−0.194
4	Italy	2	9	1	2.00	9.000	2020.7	71.00	1.09	−0.541	−0.043
5	Türkiye	4	7	2	2.00	7.000	2022.4	19.71	1.85	−0.473	−0.191
6	Canada	3	7	3	5.00	7.000	2023.3	11.86	1.24	−0.536	0.077
7	Germany	1	7	3	3.00	7.000	2023.3	37.29	1.94	−0.455	0.139
8	United Kingdom	1	6	2	2.00	6.000	2023.2	11.00	0.57	−0.410	0.135
9	Saudi Arabia	5	6	1	1.00	6.000	2023.7	13.33	0.44	2.212	−0.039
10	Australia	3	5	2	3.00	5.000	2023.4	11.00	0.86	−0.561	0.038
11	South Korea	5	5	1	1.00	5.000	2024.8	6.40	0.37	2.215	0.031

## Data Availability

No new data were created or analyzed in this study. Data sharing is not applicable to this article.
